# International Centers of Excellence for Malaria Research: Achievements of the Collaborative Network during the Past Decade

**DOI:** 10.4269/ajtmh.22-0209

**Published:** 2022-10-13

**Authors:** Malla R. Rao, B. Fenton Hall

**Affiliations:** National Institute of Allergy and Infectious Diseases, National Institutes of Health, Bethesda, Maryland

During the past two decades, multilateral global health initiatives together with endemic-country efforts fueled a massive scale-up of malaria control interventions. Despite this remarkable expansion since 2000 and associated reductions in malaria mortality and morbidity documented in many countries, the rate of decrease in malaria morbidity and mortality slowed in recent years, and consequently the WHO Global Technical Strategy 2020 goals for morbidity and mortality were not met.^1^ According to the WHO *World Malaria Report 2021*,^2^ there were an additional 14 million malaria cases and 69,000 associated deaths in 2020 compared with 2019. Even though 21 countries achieved malaria elimination and more than 10 million malaria deaths were averted during the past two decades, malaria remains endemic in 87 countries, with approximately half the world’s population still at risk. While COVID-19 shocked the world’s health systems and affected malaria control campaigns worldwide, recent increases in malaria incidence and mortality are not entirely explained by COVID-related disruptions—approximately 30% of the additional malaria deaths were not attributable to disruptions of malaria diagnosis and treatment services resulting from COVID-19.^2^

Pandemic-related disruptions are certainly not the only threat to malaria control efforts. Other well-known, major threats to malaria control include emergence and spread of drug and insecticide resistance; emergence and spread of mutated parasites that evade detection by current rapid diagnostic tests (RDTs); and the effects of natural disasters, humanitarian crises, and political unrest. In addition, however, there are new and emerging threats. Extreme weather events, now being experienced more frequently, and rising ambient temperatures caused by climate change may have implications for both the transmission capacity of malaria vectors as well as their population distribution. Changing climatic patterns are also predicted to create both water shortages and flooding in vulnerable regions, which could affect mosquito breeding habitats. In some areas, man-made modifications to the environment have increased the susceptibility of human populations to mosquito exposure^3^^,^^4^ or altered the mix of vector species in those environments.^5^ Although malaria risk is usually highest in rural settings, according to the WHO Strategic Advisory Group for malaria eradication, increasing urbanization will contribute to environmental changes that could affect the risk of malaria and the global eradication agenda.^6^ Indeed, the risk of malaria in urban areas is expected to increase because it is anticipated that 70% of the world’s population will be living in cities by 2050, with 90% of this growth occurring in Asia and Africa.^7^^,^^8^ Environmental modifications in peri-urban areas and urban slums generating vector breeding habitats, adaptation of vectors to polluted waters,^9^ movement of infected individuals from outlying endemic areas,^10^ as well as the introduction and spread of vectors capable of breeding and sustaining transmission in urban settings^11^ are all expected to be contributory factors.

All of these threats require investments in research to understand, predict, and control malaria now and in the future, if progress toward elimination and eventual eradication is to be restored and sustained.

**Figure 1.**
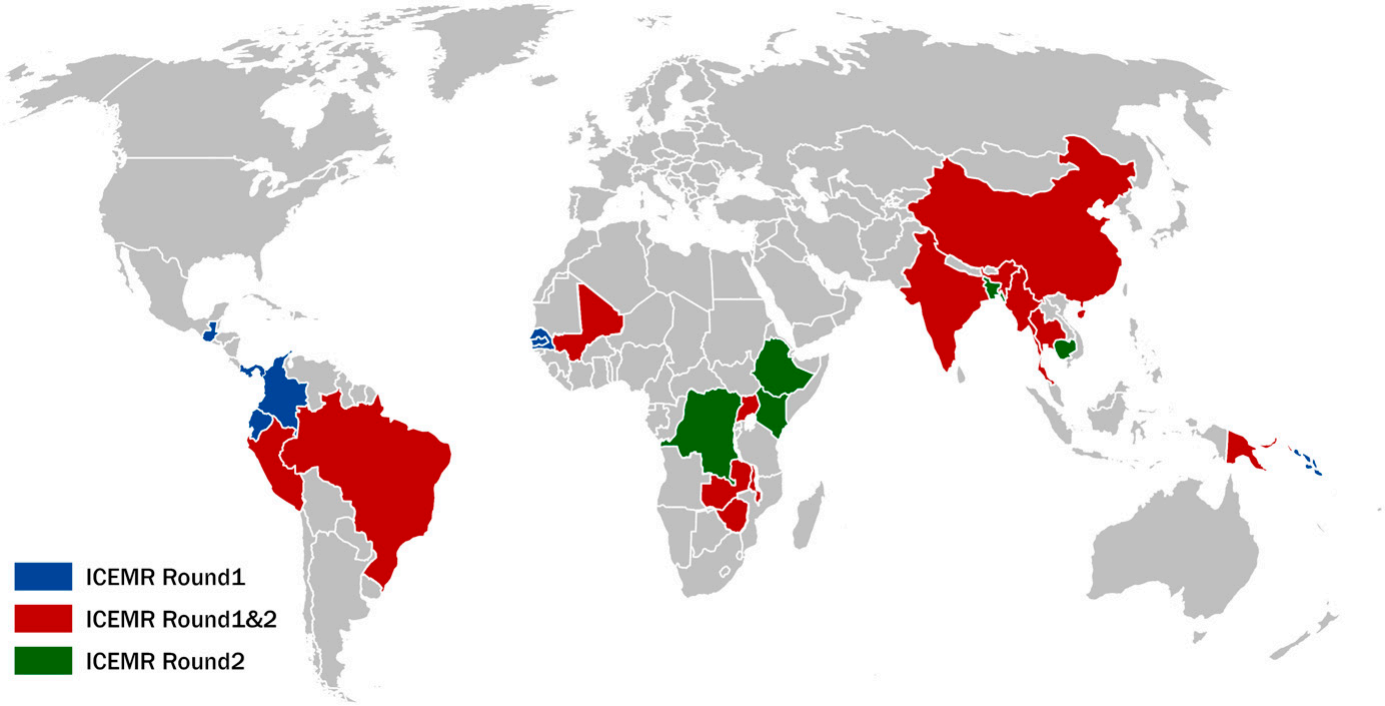
International Centers of Excellence for Malaria Research (ICEMR) countries in rounds 1 and 2. Round 1 centers established in 2010 and round 2 in 2017.

In 2010, in response to the call for improved global malaria control and elimination, the National Institute of Allergy and Infectious Diseases of the U.S. NIH established the International Centers of Excellence for Malaria Research (ICEMR) program.^12^ From the outset, the purpose of the program has been to conduct integrated multidisciplinary malaria research on the host, vector, and pathogen in different eco-epidemiological and transmission settings. Ten ICEMRs were established across all malaria-endemic continents, with each center conducting research in at least three distinct transmission settings (Figure 1). This approach was chosen to allow for the study of within-region heterogeneity and also to assess the generalizability of findings across different settings.^13^ The ICEMRs’ multidisciplinary design has enabled the centers to conduct studies and test hypotheses ranging from clinical aspects of malaria at both the patient and population levels to basic science at the molecular level. Furthermore, the ICEMRs have been able to take advantage of a 7-year funding commitment. This extended duration has enabled them to collect long-time series data to study malaria seasonality and capture the effects of control interventions. During the past decade, more than 1,000 peer-reviewed publications have acknowledged receiving ICEMR support. Selected important and illustrative findings are highlighted here.

At one hyperendemic site in Uganda, where children experienced three to five clinical episodes of malaria per year, universal distribution of long-lasting insecticide-treated nets (LLINs) alone had only a limited impact.^14^ At this same site, however, the addition of repeated rounds of indoor residual spraying (IRS) with effective insecticides resulted in almost complete elimination of episodes of clinical malaria after 5 years.^15^ Moreover, this site experienced a dramatic reduction in populations of *Anopheles gambiae *s.s. and *Anopheles funestus*, the main malaria vectors in the area.^16^ In other historically highly endemic sites in Uganda, when IRS was stopped after 5 years, within 10 months there was a 5-fold increase in the incidence of malaria; within 8 months after reinstating IRS, however, this increased incidence, however, dropped 5-fold within 8 months after reinstating IRS.^17^ Furthermore, molecular analysis of the parasites within infected individuals showed reductions in the multiplicity of infection over time, indicating fewer distinct parasite genotypes within individuals and a reduction in the diversity of the parasites at the population level (personal communication, Grant Dorsey, ICEMR, Uganda). These results suggest that effective IRS can reduce significantly both the parasite and vector reservoirs as well as parasite population diversity, but it needs to be sustained in high-transmission settings.

ICEMR studies have also uncovered a shift in the age-specific burden of malaria. Historically, the burden of malaria in sub-Saharan Africa has been greatest in children younger than 5 years of age, but recently the ICEMRs in that region have observed an increasing burden of malaria in school-age children 5 to 15 years old. ICEMR studies in the Tororo region of Uganda and in Malawi have shown that school-age children have high rates of asymptomatic infection and are an important transmission reservoir because they are highly infectious to anopheline mosquitoes.^15^^,^^18^^,^^19^ These findings have implications for control programs, such as those emphasizing school-based interventions and improved bed net use targeting school-age children.^20^

In regions with seasonal malaria, where the burden of malaria has typically been highest in children 5 years of age or younger, seasonal malaria chemoprevention (SMC) is a commonly used intervention whereby sulfadoxine–pyrimethamine with amodiaquine (SP-AQ) is administered systematically to children during the transmission season. The West Africa ICEMR recently demonstrated that the impact of this strategy on overall malaria incidence was limited when SMC was provided only to those younger than 5 years because of the high burden of malaria in the 5- to 15-year-old age group.^21^ In an implementation study in Mali, investigators demonstrated a significantly greater effectiveness of SMC by expanding the target age range to include school-age children.^21^ The ICEMR also conducted a study in collaboration with the Ministry of Health in which they compared SP-AQ to an alternative combination of dihydroartemisinin–piperaquine, which putatively has fewer side effects and the same efficacy. The study found that compared with SP-AQ, dihydroartemisinin–piperaquine had both improved compliance by study participants in taking all recommended doses as well as greater participation at the community level as a result of its better taste and fewer side effects (personal communication, Seydou Doumbia, ICEMR, Mali). These study participants are being monitored with molecular epidemiological tools to assess the emergence of drug resistance. Findings from these studies are expected to inform policymakers in Mali in adjusting SMC control strategies.

In addition to monitoring for drug resistance, ICEMRs have used molecular surveillance to discover important changes in *Plasmodium* parasite populations. These centers were among the first to report an increase in the population of histidine-rich protein 2 (HRP2)- and HRP3-negative *Plasmodium falciparum* in malaria-endemic regions.^22^ Because HRP2 and HRP3 are the antigens detected by RDTs used at most point-of-care settings in Africa, an increase in HRP2/3-negative parasites could lead to increased false-negative diagnostic results. Recent studies in the Horn of Africa suggest that in some regions more than 20% of *P. falciparum* parasites do not express the HRP2 antigen, which has led the WHO Malaria Policy Advisory Group to issue a statement recommending the use of quality assured, non-HRP2–based RDTs when the local prevalence of *Pfhrp2/3* deletions exceeds 5%.^23^ The implication of this observation is profound. Because access to health facilities is limited in many remote areas, and diagnosis using slide microscopy is often not available, community health workers typically rely on RDTs and antimalarials to detect and treat malaria cases. In areas with a high prevalence of HRP2/3-negative parasites, malaria cases may be going undiagnosed and therefore untreated. In addition to the continued surveillance of *Pfhrp2/3* deletions, the ICEMRs are conducting molecular surveillance to study the emergence and spread of antimalarial drug resistance alleles in parasites, as well as to identify and study the molecular basis for insecticide resistance in mosquitoes.

The ICEMRs have also made important observations in vector behavior and ecology. ICEMRs in Africa, Latin America, and Asia have noted a shift in the biting behavior of mosquitoes, with more mosquitoes biting outdoors and earlier in the evening, potentially reducing the overall effectiveness of long-lasting insecticide-treated nets and IRS, which act indoors.^24^ Currently, there are very few interventions that can reduce outdoor human–mosquito contact sustainably. The degree of contact can be altered through interventions that induce changes in the behavior of both mosquitoes and humans. Preliminary studies of attractant toxic sugar bait traps in Mali appear to show promising results.^25^ The environment at the Mali study site (dry climate and sparse vegetation), however, is not representative of most malaria-endemic areas, where natural vegetation may provide alternative sugar sources. Deployment of attractant toxic sugar bait traps should be investigated in areas with different vegetation sugar sources to assess and optimize their effectiveness in such areas. Other potential interventions, including the use of endectocides in humans and cattle, personal and spatial repellents, and long-lasting larvicides, need more comprehensive evaluation. More research is needed to understand temporal shifts in biting behaviors, and novel products are needed to reduce residual transmission after indoor control efforts resulting from increased outdoor biting.

At a more basic science level, using isolates from five ICEMR regions covering more than 10 countries, the ICEMRs generated the first global genetic diversity map of *Plasmodium vivax*, with isolates from five ICEMR regions, covering more than 10 countries.^26^ These maps showed distinct subpopulations, selective sweeps of drug resistance genes, and genome diversity hotspots. The observations suggest that *P. vivax* is adapting not only to regional differences in human and mosquito hosts, but also to underlying variations in the force of infection. An enhanced capacity to adapt, resulting from high genetic and functional variation, will make *P. vivax* malaria more difficult to eradicate, requiring modifications to local control methods and deeper surveys of *P. vivax* genetic variation to monitor the progress of elimination efforts.

Because of the collaborative nature of the network, the ICEMRs have been able to establish joint projects across regions, comparing research findings obtained concomitantly using similar protocols, case definitions, and technologies. As a result, it was possible to conduct comparative studies of cerebral malaria (CM) in Africa and Asia using MRI. Radiologists across continents assessed and classified independently scans of children and adults using standardized approaches. Brain magnetic resonance images and blood profiles from cohorts of pediatric and adult CM cases in India and pediatric cohorts in Malawi identified common correlates of brain swelling—namely, high parasite biomass and increased *P. falciparum *var transcripts associated with binding to host endothelial protein C receptor.^27^^,^^28^ ICEMR investigators in India also showed that brain swelling was increased in older patients with CM, and further evaluated brain volume using the apparent diffusion coefficient (ADC). Fatal CM was associated with decreased ADC in adults, suggesting cytotoxic edema, whereas in children it was associated with increased ADC, consistent with vasogenic edema. They also found brain swellings had different areas of restricted diffusion in children compared with adults. Although brain swelling was observed in approximately two thirds of children and adults, it resolved upon treatment in both populations, and scanned images of survivors exhibited similarities to the “posterior reversible encephalopathy syndrome.”^29^

By encouraging and facilitating collaboration among centers, the ICEMR program structure has enabled the centers to develop shared protocols and technologies. The ICEMRs have made efforts to harmonize outcomes and predictor variable definitions, and—to the extent possible—they have adopted Clinical Data Interchange Standards Consortium (https://www.cdisc.org/standards) standards that enable data sharing and facilitate merging of data sets. ICEMRs have also made their data sets publicly accessible via ClinEpiDB (https://clinepidb.org/) and VEuPathDB (https://veupathdb.org/)—databases that host demographic, clinical, vector, phenotypic, and genomic data. In addition to being data repositories, these resources provide access to data mining and analytical tools, thus providing a computational platform to the scientific community. Public access to these resources allows researchers and policymakers to query data across regions and view or download results immediately, obviating the need to merge data sets or struggle with the definitions of variables and their codes.^30^^,^^31^

What sets the ICEMR program apart from most malaria research programs is not only the broad scientific scope and geographic coverage, but also the extensive collaborations between ICEMRs and local ministries of health, non-governmental organizations, and other stakeholders involved in malaria research and control. Quite often, these interactions have resulted in changes to local control strategies and have spurred further linkages and collaborations among partners. The ICEMR program is also unusual in its inclusion of training of early-stage investigators (ESIs) and building research capacity in endemic-country institutions. Over the years, more than 50 ESIs have received hands-on field experience through the ICEMR network, with many going on to become principal investigators on their own NIH grants. In a recent survey by an external consultant, 81% of current and former ICEMR ESIs indicated they were “very satisfied” with the mentorship provided by the program (internal communication, NIAID). In addition to training, ICEMR collaborations have provided endemic-country institutions with valuable experience in grants administration and multidisciplinary field research methods.

For this Supplement, each ICEMR has provided two articles, the first covering some of their salient research findings and achievements during the past decade, and the second describing their engagement with in-country partners and impact on local malaria control policy. The Supplement provides an overview of the how the ICEMRs’ field research and findings have influenced various stakeholders across the spectrum from fundamental malaria research to malaria control and elimination programs.
